# Successful treatment using immunotherapy in combination with chemotherapy for metastatic squamous cell carcinoma of unknown primary origin with bulky abdominal mass: A case report

**DOI:** 10.1097/MD.0000000000028074

**Published:** 2021-12-03

**Authors:** Min Zhang, Meng Zhao, Li-fang Jin, Wei-zhang Shen

**Affiliations:** Department of Oncology and Hematology, The Second Hospital of Jilin University, Nanguan District, Changchun City, Jilin, China.

**Keywords:** cancer of unknown primary (CUP), case report, immunotherapy, metastatic squamous cell carcinoma

## Abstract

**Rationale:**

Cancer of unknown primary (CUP) means that the primary focus cannot be found after preliminary clinical evaluation. It accounts for 2.3% to 5% of newly diagnosed cancer cases. Due to the lack of standard treatment, CUP is usually associated with poor prognosis and is the third to fourth most common cause of cancer-related deaths.

**Patient concerns:**

We report the case of a 42-year-old female patient who was admitted to the hospital for intermittent right abdominal pain and abdominal distension. Abdominal computed tomography (CT) showed a large abdominal mass of unknown origin, which was difficult to resect due to its close relationship with surrounding tissues. Twenty days later, the patient had enlarged left supraclavicular lymph nodes, and percutaneous biopsy revealed squamous cell carcinoma. In addition, next-generation sequencing (NGS) of tissue and blood samples showed immune-related mutations and PD-L1 expression.

**Diagnoses:**

The patient was diagnosed with metastatic squamous cell carcinoma of unknown primary origin, with a bulky abdominal mass.

**Interventions:**

The patient was treated with carboplatin, albumin-binding paclitaxel, and immune checkpoint inhibitor (carilizumab). After 6 cycles, the patient was switched to maintenance treatment with carilizumab.

**Outcomes:**

The general condition of the patient improved, and the lesion was significantly reduced. The treatment efficacy was assessed as partial remission according to Response Evaluation Criteria in Solid Tumors. The patient benefited from immunotherapy combined with chemotherapy.

**Lessons:**

There is no recommended standard treatment for most CUPs, which leads to their poor prognoses. By performing NGS for patients and targeting immune-related positive predictors, immunotherapy combined with chemotherapy may prolong the overall survival of patients. This case report suggests that immunotherapy combined with chemotherapy is feasible and effective in patients with CUP.

## 1 Introduction

Cancer of unknown primary (CUP) is diagnosed in a group of malignant tumors confirmed by pathological biopsy; however, the location of the primary tumor cannot be determined by clinical physical examination, laboratory tests, and routine imaging.^[1]^ Worldwide, CUP is the sixth to eighth most common malignant tumor accounting for 2.3% to 5% of new cancer diagnoses, and only 15% to 20% of patients with CUPs can be treated similarly to patients with equivalent known primary focus with metastatic dissemination. However, because most patients cannot be recommended a standard treatment, the prognosis is poor, making CUP the third to fourth most common cause of cancer-related death.^[2,3]^ We, herein, present the case of a CUP patient with a bulky abdominal mass and metastatic squamous cell carcinoma (SCC) who benefited from immunotherapy combined with chemotherapy, and we review the literature on the topic.

## 2 Case report

**Figure 1:**
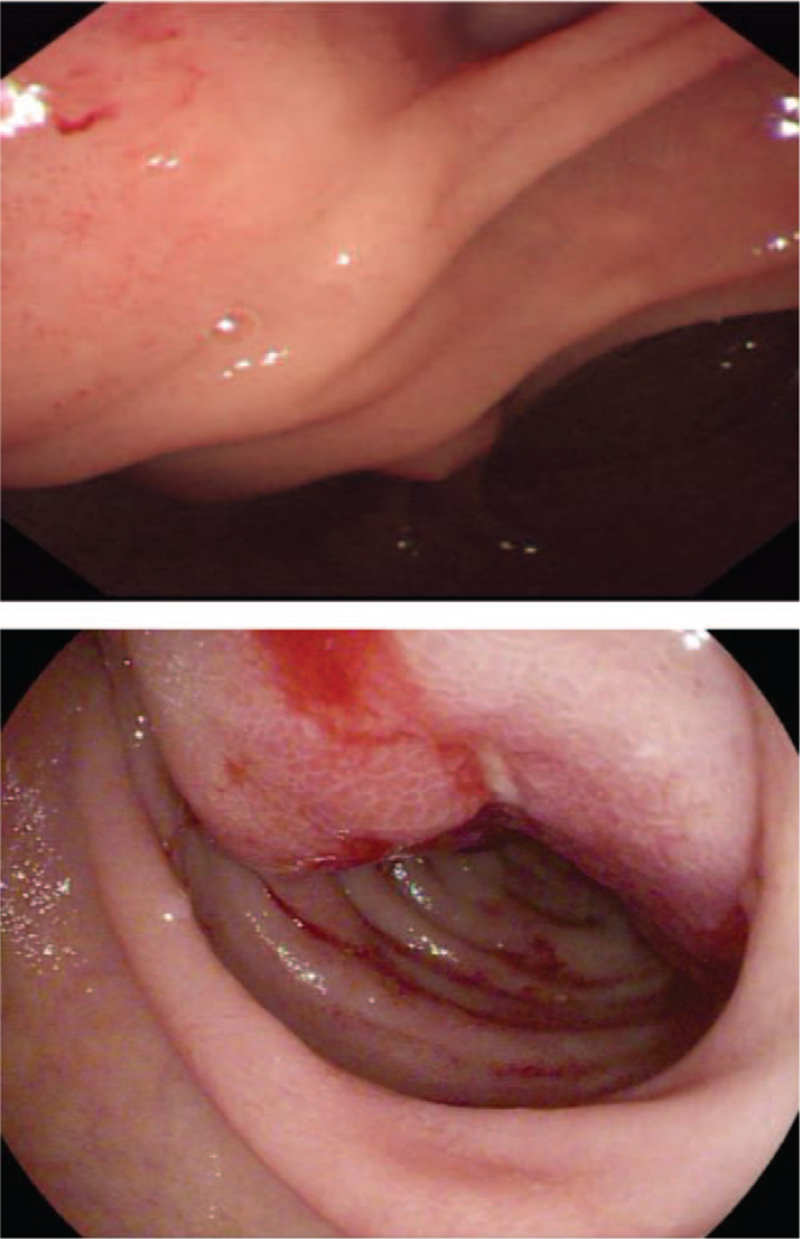
Gastroduodenoscopy revealed a large mass at the junction of the bulb and the descending portion of the duodenum.

A 42-year-old female patient was admitted to the hospital for a 3-month history of intermittent right abdominal pain, abdominal distension, loss of appetite, and weight loss of 10 kg. Two years ago, she underwent cholecystectomy and left liver lobectomy due to calculus of the intrahepatic duct and atrophy of the left liver. On examination by her physician, she was conscious and without jaundice or scleral icterus. She had mild epigastric tenderness on abdominal examination, and there was no superficial lymph node enlargement. Laboratory examination showed that leukocyte levels were 14.7 × 10^9/L (normal: 3.5–9.5 × 10^9/L), neutrophil levels were 12.57 × 10^9/L (normal: 1.8–6.3 × 10^9/L), and serum electrolytes and renal and liver functions were within normal limits. The serum albumin level was 36.4 g/L (normal: 40–55 g/L), and the lactate dehydrogenase (LDH) level was 317 U/L (normal: 120–250 U/L). Laboratory tests showed elevated tumor markers, with a CA153 level of 42.8 U/mL (normal: 0–31.3 U/mL), SCC antigen level of 3.6 ng/mL (normal: 0–1.5 ng/mL), and cytokeratin fragment level of 94 ng/mL (normal: 0–2.08 ng/mL), whereas the rest of the biochemical parameters were all normal. The chest computed tomography (CT) showed no abnormalities, while the abdominal CT enhancement scan showed a soft tissue density mass of approximately 56 × 46 mm in the medial part of the descending duodenum with uneven enhancement, along with multiple lymph node enlargements in the abdominal cavity and retroperitoneum. Gastroduodenoscopy revealed a large mass at the junction of the bulb and the descending portion of the duodenum (Fig. 1); the surface is uneven, brittle texture, and easy to bleed. Pathological examination of the tumor showed chronic mucosal inflammation, as well as a small number of heterocysts in the local lamina propria.

Because of the symptoms of intestinal obstruction, the patient agreed to undergo surgical treatment after a multidisciplinary discussion. During the operation, it was found that the tumor could not be separated from the retroperitoneum and hepatic porta, and the fusion lymph nodes near the abdominal aorta surrounded the superior mesenteric artery; thus, removal of the tumor was difficult. After discussion, it was decided to temporarily solve the problem of incomplete obstruction caused by the tumor pressing on the duodenum, and the patient agreed to undergo distal gastrectomy and gastrointestinal anastomosis. The surgeon did not take specimens for pathology at the primary lesion. However, distal gastrectomy postoperative pathology showed chronic mucosal inflammation with expansion and congestion of the subserosal vessels, which could be seen as acute inflammatory exudation in local areas. The type of tumor remained unclear.

**Figure 2:**
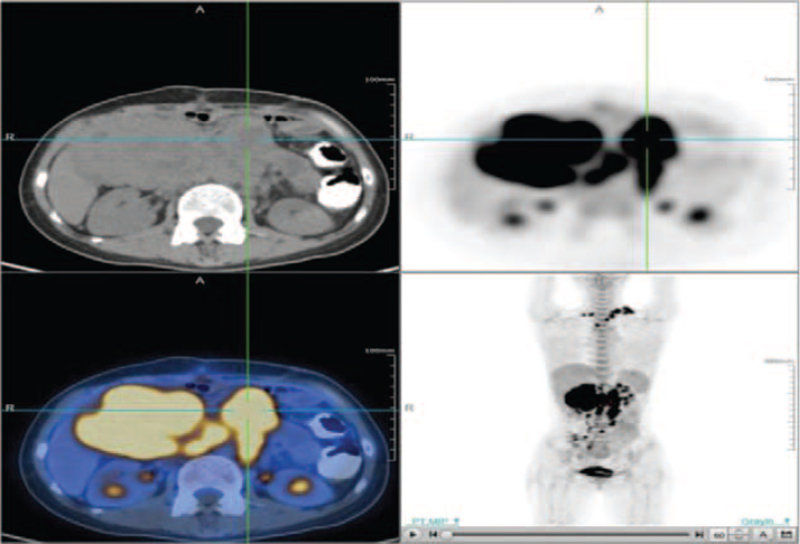
PET/CT showed a large duodenal mass with strong FDG uptake and multiple lymph node metastases.

**Figure 3:**
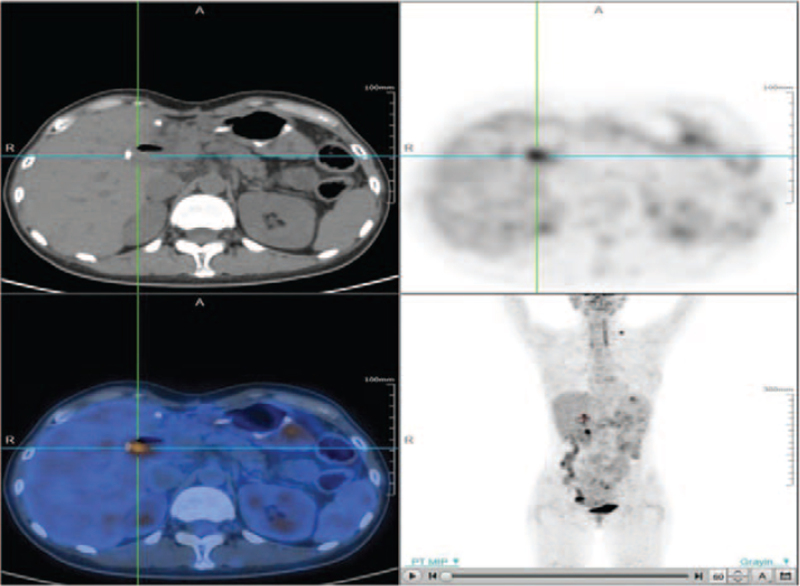
After 6 cycles of immunotherapy combined with chemotherapy, PET/CT showed that the tumor was significantly reduced.

However, 20 days after the operation, the left supraclavicular lymph nodes were found to be enlarged, and percutaneous biopsy revealed SCC. Next-generation sequencing (NGS) of tissue and blood samples showed G13D [c.38G>A (p.G13D)) KRAS mutations, TP53 (c.919+1G>A) mutations, S941 (c.2822C>A (p.S941∗)) PBRM1 mutations, T976N (c.2387C>A (p.T976N)] MSH2 mutations, and a low mutational load (3.2/Mb). Immunohistochemistry showed that PD-L1 protein expression in a metastasis representative was 20%. The 18F-2-fluoro-2-deoxy-D-glucose (18F-FDG) positron emission tomography/CT (PET/CT) imaging revealed a 77.3×55.4 mm mass with intense FDG uptake in the duodenal region, which is the unclear boundary with the duodenum (the maximum of standard uptake value = 25.31, Fig. 2). Multiple lymph nodes with increased FDG uptake were found in the left posterior cervical space, bilateral clavicular area, mediastinum, left pectoral major, axilla, hepato-gastric space, mesentery, bilateral phrenic foot, retroperitoneal abdominal aorta, vena cava, and bilateral iliac total, and the largest one measured approximately 38.5 × 28.7 mm (the maximum of standard uptake value = 28.7). The patient was diagnosed with metastatic SCC of unknown primary origin with a bulky abdominal mass. According to Spanish Society of Medical Oncology (SEOM) clinical guidelines, SCC can be treated with paclitaxel [175 mg/m^2^, day (D)1] and carboplatin (5AUC, D1) at intervals of 21 days.^[3]^ In addition, NGS has shown that immune-related mutations (TP53, KRAS, PBRM1 mutations) with PD-L1 expression may indicate the efficacy of immunotherapy. Studies have found that the efficacy of albumin-binding paclitaxel plus carboplatin is better than that of paclitaxel plus carboplatin in SCC, and the toxicity can be tolerated.^[4]^ Therefore, the patient was treated with carboplatin (500 mg, D1), albumin-binding paclitaxel (180 mg, D1, D8), and immune checkpoint inhibitor (carilizumab, 200 mg, D1), 21 days per cycle. Treatment was well tolerated and could be administered without serious side effects. The patient benefited significantly after 6 cycles of treatment; PET imaging showed that the lesion was significantly smaller than before, and the size was approximately 12.3 × 10.9 mm (the maximum of standard uptake value = 7.23, Fig. 3). Moreover, lymph nodes decreased in size and partially disappeared, and FDG uptake decreased to a normal level. No abnormalities were detected in the hematological and biochemical parameters. Clinically, the general condition of the patient improved, and tumor-related symptoms, such as abdominal pain, completely disappeared. The treatment's efficacy was assessed as partial remission according to Response Evaluation Criteria in Solid Tumors. After 6 cycles, the regimen was switched to maintenance treatment with carilizumab (200 mg, D1, intervals of 21 days). At the time of writing, the patient was receiving the fifth cycle of carilizumab maintenance therapy, the focus was stable, and the patient remained in good condition with no immune-related adverse reactions.

## 3 Discussion

Currently, 2 predominant theories exist regarding the mechanism of CUP: one is that CUP is a metastatic tumor originating from a small, undetectable, or regressed primary lesion, while others argue that CUP is a single metastatic entity with no primary tumor present and is therefore biologically distinct from other metastatic tumors.^[5]^ The first examination to confirm CUP should include a comprehensive physical examination, basic blood and biochemical analysis, histological examination with immunohistochemistry staining, CT of the chest, abdomen, and pelvis, and endoscopic examination if necessary.^[6]^ The role of PET testing in identifying primary sites missed by conventional imaging and previously undiagnosed metastases has also been recognized, especially in head and neck cancer.^[7]^ Gatalica et al^[8]^ found that in 96% of cases, the use of immunohistochemistry, gene sequencing, and in situ hybridization may provide more accurate drug selection for CUP and, consequently, improve the survival of the patients.

According to SEOM clinical guidelines,^[3]^ SCC only accounts for 5% of CUP patients, and patients were divided into favorable and unfavorable groups. Patients in the favorable group can be treated similarly to patients with equivalent known primary focus and have a better prognosis. On the contrary, the unfavorable subgroups account for 80% of cases and have the characteristics of visceral disease, high tumor burden, and short survival time.^[5]^ Unfavorable groups do not have a standard treatment; therefore, doublets with platinum may be a reasonable choice.^[3]^ Most patients received platinum- and taxane-based therapy with a response rate of 15% to 20% and a mean survival time of 9 months.^[3,9]^ Studies have shown that performance status (PS), liver involvement, and LDH level are also associated with prognosis.^[10]^ The latest research also found that inferior prognosis was associated with KRAS activation by point mutation and gene amplification, and TP53 mutations were associated with an adverse prognosis in the SCC subgroup and in females.^[11]^

The PS score of our patient was 1, her baseline LDH was elevated, the abdominal lesion was large and grew fast, and she belonged to the unfavorable group. TP53 and KRAS mutations all suggest a poor prognosis. Therefore, this patient is unlikely to benefit from chemotherapy alone. We know that CUP patients with PD-L1 expression, high TMB, and MSI-H can benefit from immune checkpoint inhibitor treatment.^[12]^ Moreover, a recent study also found that TP53 and KRAS mutation status and PBRM1 mutation can benefit from anti-PD-1 /PD-L1 immunotherapy^[13,14]^; it may benefit those with CUP as well. Therefore, we added the immune checkpoint inhibitor anti-PD1 carilizumab. After chemotherapy and immunotherapy, the patient's lesions were significantly reduced, her general state improved, and her PS score was 0. The patient benefited significantly.

Stefan Groschel et al also reported the benefit of immune checkpoint inhibitors in a case of refractory CUP with PD-L1 amplification and overexpression.^[15]^ In addition, according to patients’ NGS, some clinical studies have reported benefits from CUP-targeted therapy,^[16]^ including partial response in CUP patients with KRAS (G12D) mutations treated with Trimetini (MEK inhibitors), and complete clinical response in patients with CUP treated with BRAF (V600E) targeted therapy with vemurafenib combined with immunotherapy drug ipilimumab. The Phase II global CUP trial (NCT03498521), which is expected to be completed by June 30, 2023, will further evaluate the progress of targeted therapy and immunotherapy in CUP patients and may contribute to the discovery of novel biomarkers.

## Author contributions

**Conceptualization:** Min Zhang, Wei-zhang Shen.

**Data curation:** Meng Zhao, Li-fang Jin.

**Investigation:** Meng Zhao, Li-fang Jin.

**Writing – original draft:** Min Zhang.

**Writing – review & editing:** Wei-zhang Shen.

## References

[1] Uzunoglu S Erdogan B Kodaz H. Unknown primary adenocarcinomas: a single-center experience. Bosn J Basic Med Sci 2016;16:292–7. 27455119 10.17305/bjbms.2016.1495 PMC5136766

[2] Qaseem A Usman N Jayaraj JS. Cancer of unknown primary: a review on Clinical Guidelines in the Development and Targeted Management of Patients with the Unknown Primary Site. Cureus 2019;11:e5552. 31695975 10.7759/cureus.5552 PMC6820325

[3] Losa F Soler G Casado A. SEOM clinical guideline on unknown primary cancer (2017). Clin Transl Oncol 2018;20:89–96. 29230692 10.1007/s12094-017-1807-y PMC5785607

[4] Wang H Mou S Tu M. Study on the effect of nano albumin paclitaxel combined with carboplatin in the treatment of lung squamous cell carcinoma. J Nanosci Nanotechnol 2020;20:7439–43. 32711612 10.1166/jnn.2020.18880

[5] Conway AM Mitchell C Kilgour E. Molecular characterisation and liquid biomarkers in Carcinoma of Unknown Primary (CUP): taking the ’U’ out of ’CUP’. Br J Cancer 2019;120:141–53. 30580378 10.1038/s41416-018-0332-2 PMC6342985

[6] Pauli C Bochtler T Mileshkin L. A challenging task: identifying patients with cancer of unknown primary (CUP) according to ESMO Guidelines: the CUPISCO Trial Experience. Oncologist 2021;26:e769–79. 33687747 10.1002/onco.13744 PMC8100559

[7] Wong B Vickers MM Wheatley-Price P. The diminishing importance of primary site identification in cancer of unknown primary: a Canadian single-center experience. Front Oncol 2021;11:634563. 33747958 10.3389/fonc.2021.634563 PMC7968101

[8] Gatalica Z Millis SZ Vranic S. Comprehensive tumor profiling identifies numerous biomarkers of drug response in cancers of unknown primary site: analysis of 1806 cases. Oncotarget 2014;5:12440–7. 25415047 10.18632/oncotarget.2574 PMC4322997

[9] Yoon HH Foster NR Meyers JP. Gene expression profiling identifies responsive patients with cancer of unknown primary treated with carboplatin, paclitaxel, and everolimus: NCCTG N0871 (alliance). Ann Oncol 2016;27:339–44. 26578722 10.1093/annonc/mdv543 PMC4907341

[10] Petrakis D Pentheroudakis G Voulgaris E Pavlidis N. Prognostication in cancer of unknown primary (CUP): development of a prognostic algorithm in 311 cases and review of the literature. Cancer Treat Rev 2013;39:701–8. 23566573 10.1016/j.ctrv.2013.03.001

[11] Bochtler T Reiling A Endris V. Integrated clinicomolecular characterization identifies RAS activation and CDKN2A deletion as independent adverse prognostic factors in cancer of unknown primary. Int J Cancer 2020;146:3053–64. 31970771 10.1002/ijc.32882

[12] Raghav K Overman M Poage GM. Defining a distinct immunotherapy eligible subset of patients with cancer of unknown primary using gene expression profiling with the 92-gene assay. Oncologist 2020;25:e1807–11. 32893931 10.1634/theoncologist.2020-0234 PMC7648339

[13] Dong ZY Zhong WZ Zhang XC. Potential predictive value of TP53 and KRAS mutation status for response to PD-1 blockade immunotherapy in lung adenocarcinoma. Clin Cancer Res 2017;23:3012–24. 28039262 10.1158/1078-0432.CCR-16-2554

[14] Aili A Wen J Xue L Wang J. Mutational analysis of PBRM1 and significance of PBRM1 mutation in anti-PD-1 immunotherapy of clear cell renal cell carcinoma. Front Oncol 2021;11:712765. 34447697 10.3389/fonc.2021.712765 PMC8383204

[15] Gröschel S Bommer M Hutter B. Integration of genomics and histology revises diagnosis and enables effective therapy of refractory cancer of unknown primary with PDL1 amplification. Cold Spring Harb Mol Case Stud 2016;2:a001180. 27900363 10.1101/mcs.a001180 PMC5111004

[16] Es HA Mahdizadeh H Asl AAH Totonchi M. Genomic alterations and possible druggable mutations in carcinoma of unknown primary (CUP). Sci Rep 2021;11:15112. 34302033 10.1038/s41598-021-94678-4 PMC8302572

